# Influence of the Punch Shape on Formability Measurement During Dry Fabric Preforming

**DOI:** 10.3390/ma18245535

**Published:** 2025-12-09

**Authors:** Rym Azzouz, Samir Allaoui

**Affiliations:** Institut de Thermique Mécanique Matériaux, Université de Reims Champagne Ardenne (ITheMM UR7548), Campus Sup Ardenne, 9A Rue Claude Chrétien, 08000 Charleville-Mézières, France; samir.allaoui@univ-reims.fr

**Keywords:** defects, forming, preform, fabrics/textiles

## Abstract

The formability of reinforcement is essential for controlling shaping processes and assessing their suitability for industrial applications. The complexity of the geometries dictates the deformation modes and thus the reinforcements’ behaviours. This study is an experimental campaign to investigate the shaping of five different geometries with three reinforcements that have varying meso-structures: plain weave, interlock and Non-Crimp Fabric. The comparison concentrates on shear behaviour and defects induced. The measured parameters are chosen in relation to their potential impact on the composite’s properties at both local and macro levels. The findings reveal that geometry significantly influences the quality of the preform. Each geometry shows unique behaviours due to a different, but limited, range of mechanisms. This highlights the importance of identifying and analysing the interesting parts of these geometries and their role in triggering the different behaviours.

## 1. Introduction

The increasing demand for composite materials in industrial applications is due to their numerous advantageous properties, emphasising the importance of mastering the manufacturing process. Various processes are employed in the production of composites, each offering benefits, including liquid composite moulding processes (LCM). They provide a good production rate-to-cost ratio and facilitate the manufacturing of complex-shaped parts [1]. LCM requires a preforming of the dry reinforcement before the matrix is introduced. Preforming is critical because it can lead to fabric meso-architecture variation that disturbs material homogeneity. This is regarded as a defect once it reaches a certain severity. Studies have, therefore, focused on proposing solutions to prevent these defects [2]. Several factors are considered to optimise the preforming process, as they are significant in dictating the stress and strain distribution. As a result, the location and severity of the defects.

Formability is a property of the reinforcement, reflecting its ability to deform and adapt to a specific shape in response to various deformation modes. The limits of this property determine the quality of a preform. Therefore, for a given application, the outcome depends on three main factors: the first is the type of reinforcement, such as the material, structure, and number of layers; the second is the preforming process, including the process type and parameters (pressure, tension, speed); and the third is the punch geometry [3], including details such as the edges’ radii [4], the inclination [5], the curvatures [6], etc. The complexity of the geometry raises the risk of defects, resulting in unacceptable preform quality. The fabric is expected to deform to conform to the shape of the geometry [7]. Hence, the fabric undergoes one or more combined deformation modes, depending on its shape characteristics. Various punch geometries have been employed in the literature to examine the effects of different parameters during preforming. They are mainly inspired by metal forming, a typical repetitive detail derived from industrial applications, or a fully functional component.

For a long time, composite-forming studies have used the hemisphere [8,9,10] as the most common choice of shape, influenced by metal forming studies. The initial numerical studies using this geometry for composite preforming focused solely on the fabric’s geometrical properties and did not consider mechanical behaviour [11,12].

Later, mechanical behaviours were incorporated to enhance the numerical models [13,14,15]. This enhanced the models’ robustness, yielding results that closely match experimental data and thus predict defects such as wrinkles [16,17], buckles [16], folds and sliding [18]. The orientation of the fabric for the hemisphere is irrelevant because the hemisphere is symmetrical in any position. For woven fabrics, the hemisphere causes variations in shearing because the warps and wefts must adapt to the decreasing radii from the top of the hemisphere to its base. The double dome is a second geometric form inspired by the metal forming process. It shares many similarities with the hemisphere [19,20]. Shearing is observed in hemispherical parts. The difference lies in the cylindrical section of this shape, where slippage is likely to occur on a large scale.

Recently, new shapes, such as the tetrahedron [21,22,23], the prism [2,24,25], the cube [2,24,26] and the cone [27], are being increasingly used. These shapes reveal different and more severe behaviours not previously highlighted by the hemisphere and double dome. Some studies have explored variations or combinations of these geometries to investigate their effect on forming quality and whether they involve more mechanical behaviours and defects across a wide range of amplitudes. Some examples include a combination of the double dome and the prism geometries [20,28], or the modified tetrahedron and square box [29]. The behaviours and defects develop with geometric characteristics, highlighting the need to consider them when optimising the quality of the preform. It is complex to compare several preforms that do not share similar geometric details or dimensions. These geometries are basic shapes, generally simplified and non-functional, and are used to isolate a geometric detail for study, whereas those resulting from industrial applications aim to optimise the targeted process [2,30,31].

So, which geometries should be chosen to examine the formability of a fabric or to compare several reinforcements for selecting the most suitable one for a specific application? The choice of geometry must be based on its ability to promote a wide range of behaviours and defects, thereby allowing discrimination between different reinforcements. Many geometries and fabrics have been employed to study the behaviour during forming. However, utilising the results of previously published studies is challenging, as most were conducted with a single geometry and involved different configurations (parameters, devices, dimensional scales, and fabrics).

Each geometry triggered different behaviours and defects, prompting the authors to explore various geometries. Since no single shape or set of shapes had been identified to observe a wide range of defects and behaviours, this led to a diverse selection of shapes. Studies emphasised the need to use different geometries [32]. For the first time, this problem was highlighted in [3,23,26] using several geometric shapes with a single reinforcement, demonstrating the influence of geometry on changes in shear and the formation of wrinkling defects. J. Huang et al. [33] conducted preforming on two geometries (Cube and Hemisphere) on two woven reinforcements with a different structure. The study, which did not quantify the behaviours and defects because its objective was to validate the proposed numerical model, concluded that different types of reinforcements are needed. This aspect was later studied by R. Bai et al. [34] Eleven types of reinforcements were used during an experimental campaign involving two geometries to correlate the quality of the preform with an indicator of formability, “DR”. This indicator, which combines shear and bending stiffness, aims to predict the development of macroscopic wrinkles. They demonstrated that the higher the proposed indicator, the greater the risk and severity of wrinkles.

Furthermore, the qualitative experimental measurements only focused on shear and wrinkling. As a result, the influence of geometry and its relationship with reinforcement architecture, which exhibits a wide variety of behaviours and defects, was only briefly addressed. Therefore, it is not yet fully understood.

This study aims to contribute to this area through an experimental campaign using various types of reinforcements and geometries, focusing on several behaviours and defects during shaping. The goal is to understand how the details of the geometry influence the process. All geometries underwent the same initial preforming to isolate their effects, followed by detailed quantitative measurements. Indicators were proposed to assess both the quantitative and qualitative reinforcement behaviours, reflecting the magnitude and severity of defects. These defects were then correlated with specific geometric features to identify those that display more significant behaviours for each geometry, which could serve as a reference for studying reinforcement behaviour.

## 2. Materials and Methods

The preforming was conducted under identical conditions for all shapes to compare behaviours and defects for each geometry. The reinforcements were chosen with various structures to cover a broad spectrum of fabric types and observe the results. The geometries selected were chosen from the literature.

### 2.1. Punch Geometries

**Figure 1:**
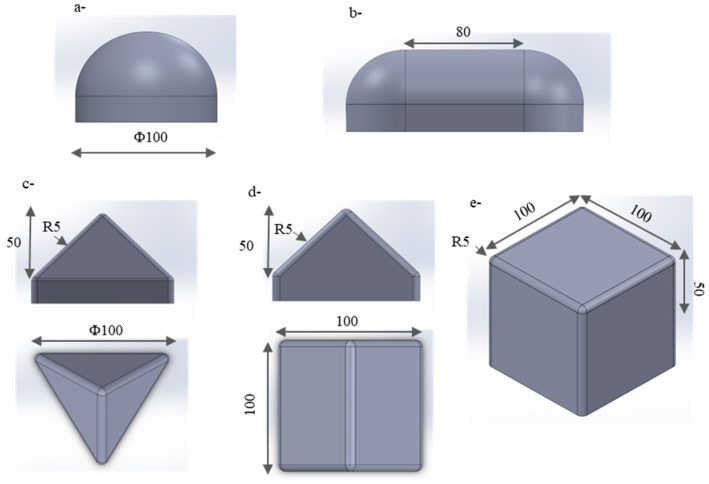
Five Punch geometries and dimensions (mm): (**a**) Hemisphere, (**b**) Double dome, (**c**) Tetrahedron, (**d**) Prism, (**e**) Cube. Adapted from Ref. [35].

Based on previous studies, five punch geometries were selected: the hemisphere, the double dome, the cube, the tetrahedron, and the prism. The hemisphere has a diameter of 100 mm (Figure 1a), and the double dome consists of two quarter-spheres, each 80 mm in diameter, spaced 80 mm apart (Figure 1b). These two shapes are the most commonly used to study forming behaviours. The other three shapes subject the fabric to severe deformation modes and are most frequently employed in the literature for complex shaping. They all share a common feature: a triple point at the intersection of three planes. The triple point forms a 90° angle at the four corners of the cube, the two tops of the prism, and the top of the tetrahedron. Each triple point has a different relative position to the reinforcement, which induces different deformation modes in the reinforcement. All the preforms were conducted at a depth of 50 mm (Figure 1). Every edge of the three geometries was given a 5 mm radius fillet.

### 2.2. The Preforming Device

**Figure 2:**
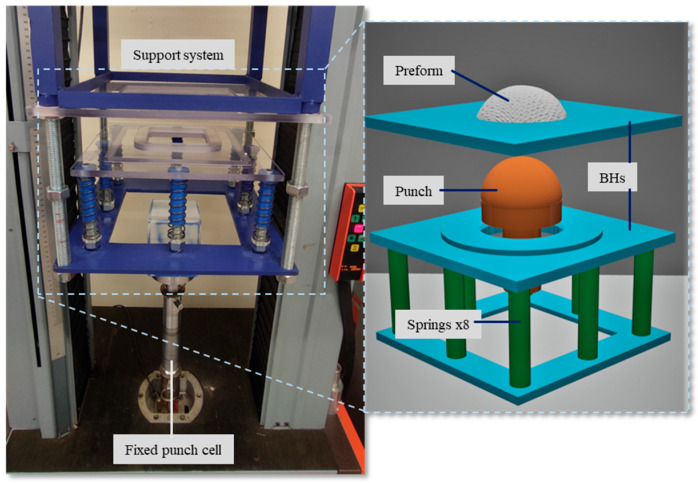
Preforming device. Adapted from Ref. [35].

The device (Figure 2) is a locally designed preforming bench mounted on a universal machine. It consists of two parts: the first is the punch attached to the machine’s fixed crosshead, while the second comprises an open die fixed on the mobile crosshead, equipped with a system to maintain fabric positioning and apply pressure from the blank holders (BHs). Pressure is applied on the fabric via eight springs evenly distributed around the perimeter of the BH to ensure uniform pressure. The BHs surrounding the entire preform contact the fabric over a surface matching the external shape of the punch. This system moves vertically during the process.

### 2.3. Fabrics

Three distinct types of dry reinforcements, covering different fabric families, were used in the study (Table 1). The three textile reinforcements have different meso-architectures but a similar areal weight. The first is a balanced glass plain weave produced by Chomarat, Le Cheylard, France, commercially referred to by the manufacturer as G-WEAVE 600p. It has an areal weight of 600 g/m^2^ ± 5%, a thickness of 0.55 mm, a warp and weft yarn count of 600 Tex, and a unit cell of 8 mm × 8 mm. The second reinforcement is carbon interlock fabric produced by EXEL Company (Épernay, France), commercially labelled G1151 by the producer. It has an areal weight of 630 g/m^2^ and is composed of T300JB 6K carbon yarns, with approximate warp and weft widths of 2 mm and 3 mm, respectively. The weaving process links three layers of weft yarns, resulting in a fabric thickness of 0.62 mm. The unit cell measures 20 mm × 15 mm, with six warp yarns and 15 weft yarns, and a nominal construction of 7.5 yarns/cm for the warp and 7.4 yarns/cm for the weft. The third reinforcement is a carbon fibre NCF (Non-Crimp Fabric) supplied by Chomarat, marketed by the manufacturer under the name BX600. It has an areal weight of 600 g/m^2^, a unit cell of 5 mm × 9 mm, and a thickness of 0.8 mm.

### 2.4. Experimental Process and Parameters

Process parameters and settings are important factors in fabric-forming results. A low BH pressure promotes the appearance of wrinkles [16]. While in-plane tension can prevent it, generating high in-plane tensile stress may lead to sliding or, in extreme cases, breakage of the fibres. Preliminary tests were conducted to determine optimal testing conditions, ensuring extreme factors did not interfere with measurements. The aim was to replicate standard industry conditions and to avoid wrinkles, thereby allowing the study to focus on how geometry affects the preforming of the reinforcements. This preliminary study fixed the settings for all configurations tested. Consequently, the BH pressure was set at 0.025 bar and the velocity at 30 mm/min. The depth of preforming was 50 mm for each.

After the preforming, a fixing resin was sprayed onto the fabric to maintain its shape for the necessary post-preforming measurements. At least three tests were performed for each configuration. The orientation of the fabric is based on the 0/90° directions (corresponding, respectively, to the warp and weft directions; for the NCF, they represent the direction of each layer, with the top layer being at 0°). The orientations were chosen so that the 0° direction of all fabrics was positioned relative to the punches following the symmetry plane of each geometry. The orientation was selected so that at least one symmetry plane of the geometry aligned with the 0° direction on the fabric, as in the triple points. Changing the orientation will lead to different outcomes.

The behaviour measurement concentrates on shearing, which was conducted by transferring the fabric pattern and the yarns’ trajectories into transparent layers to measure the angles, even on non-planar surfaces, accurately. The preforms were 3D scanned to assess buckling.

A laser 3D scanner was used to scan the outer surface of the preform. Then, using the same spatial reference, the preform was removed to scan the outer surface of the punch, allowing us to obtain the reconstructed surfaces of both. The distance between them is determined by projecting the fabric surface onto the reference surface of the punch. This process produced a map showing the variations in distance, highlighting the thickness variations caused by buckling.

Local images were captured to observe pattern changes caused by sliding. Specific measurement parameters were established for each defect to quantify it, link each geometric detail, and compare the behaviour of each reinforcement.

## 3. Results and Discussion

Shearing is the variation in the rotation angle between the warp and the weft at their intersection points; it is related to the fabric’s ability to deform in-plane. The lower the shearing rigidity, the greater the drapability, which is the capacity to take shape, and it indicates a higher risk of wrinkling [17]. The shearing angle was observed at various locations, with a focus on the highest. Regarding the defects, each is represented by a parameter indicating severity, the critical area of greatest concern, and a quantitative parameter to assess the overall damage.

### 3.1. Shearing

**Figure 3:**
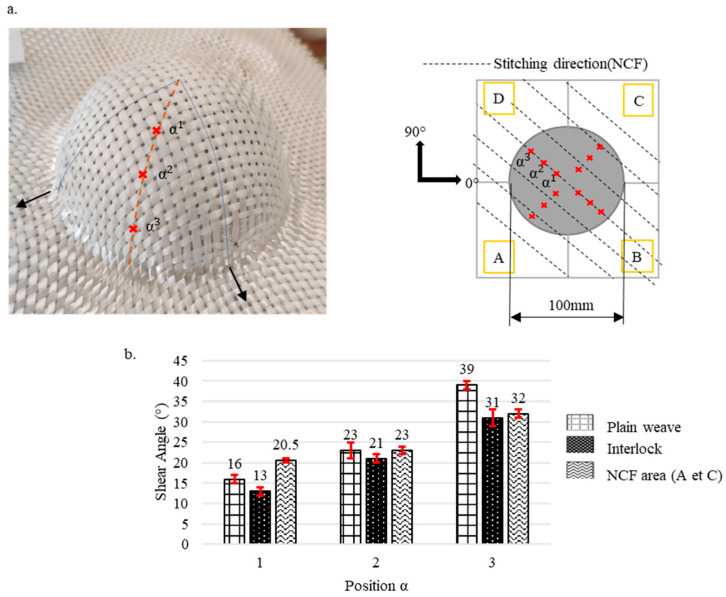
Shearing on the Hemisphere preforms for the plain weave, Interlock and NCF fabrics. (**a**) Location of the measurements. (**b**) Maximum shear angle evolution with standard deviations. Adapted from Ref. [35].

**Figure 4:**
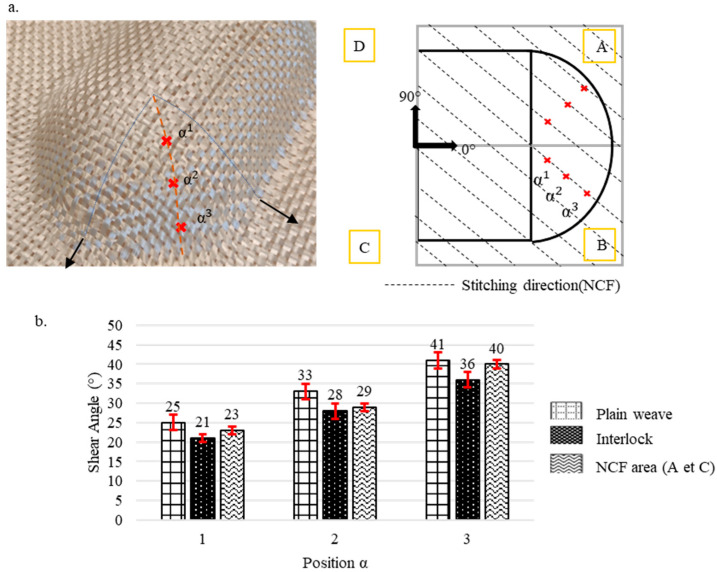
Shearing on the double dome preforms for the plain weave, Interlock, and NCF fabrics. (**a**) The location of the measurements. (**b**) Maximum shear angle evolution with standard deviations. Adapted from Ref. [35].

**Figure 5:**
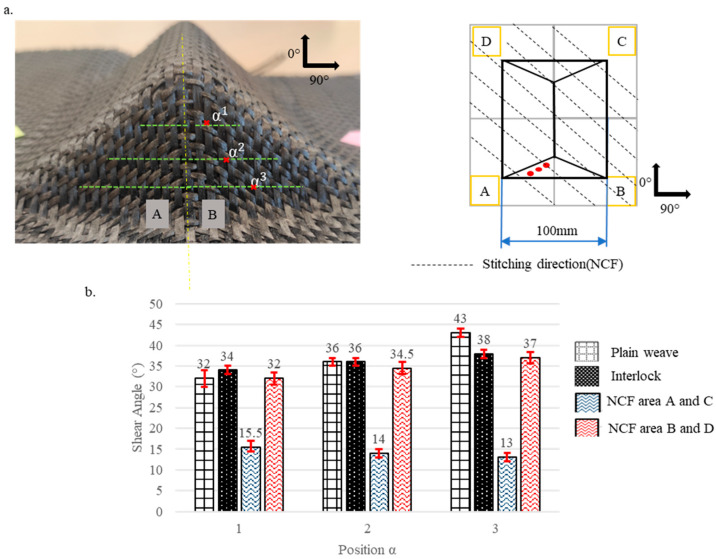
Shearing on the Prism preforms for the plain weave, Interlock, and NCF fabrics. (**a**) Location of the measurements. (**b**) Maximum shear angle evolution with standard deviations. Adapted from Ref. [35].

**Figure 6:**
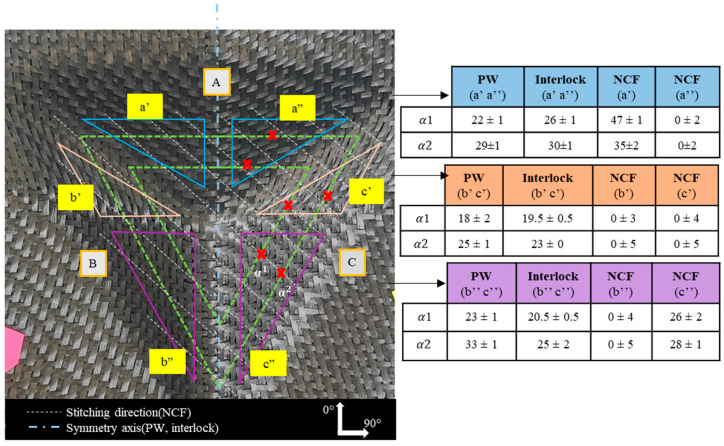
Shearing on the tetrahedron preforms for the plain weave, Interlock and NCF fabrics. Adapted from Ref. [35].

**Figure 7:**
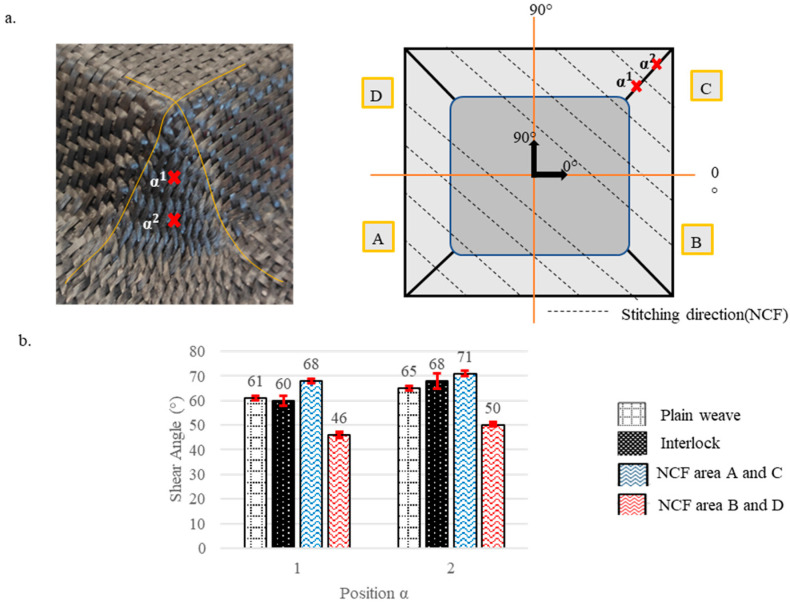
Shearing on the cube preforms for the plain weave, Interlock and NCF fabrics. (**a**) The location of the measurements. (**b**) Maximum shear angle evolution with standard deviations. Adapted from Ref. [35].

Pre-recognising that shear angles vary according to the preform’s location, each preform was divided into several zones, starting from the highest contact plane between the punch and the fabric down to the lowest depth. The levels of these zones are illustrated by dotted lines in Figure 3, Figure 4, Figure 5, Figure 6 and Figure 7. Measurements were taken at points marked as α1, α2, and α3, respectively, beginning from the top, and are shown by a red mark in the figures. The aim is to compare the shear evolution caused by each punch on each reinforcement. Therefore, these measurements were only conducted on specific faces and did not include areas with minimal shear (angles close to zero).

Additionally, zones with defects were avoided during the measurements to ensure more consistent results. The highest shear angles for each level are shown in both table and graph form (Figure 3, Figure 4, Figure 5, Figure 6 and Figure 7), where the top view of each preform highlights the measurement positions. The relative orientation of the reinforcements is indicated on the Figures by the axes 0° and 90°, corresponding to the warp and weft networks of the woven fabrics. The 0° angle represents the direction of the top-layer fibre for the NCF. The preforms were divided into four sections, labelled A, B, C, and D (Figure 3, Figure 4, Figure 5, Figure 6 and Figure 7), and A, B, and C for the tetrahedron (Figure 6), to improve readability. All values represent the average shear angles, with a maximum dispersion of 1° to 2°.

The variation in the shearing angle for the hemisphere and the double dome (Figure 3a and Figure 4a) increases from top to bottom, reaching maximum values at the bottom of the preforms (Figure 3b and Figure 4b). The high shear at the bottom of the preform is caused by the layer’s excess length, which forces the reinforcement to shear more to conform to the shape. The maximum shear for the hemisphere is the highest of the three levels at the bottom, with 39°, 31°, and 32°, respectively, for the plain weave, Interlock, and NCF (Figure 3b). These maximum values align with the literature for this type of fabric meso-architecture [23,36]. They were measured in the different zones (A to D) of the preforms for the woven reinforcements, with symmetry in the values and their evolution.

In contrast, the NCF showed an asymmetrical behaviour as observed in the literature [23,26,37]. The maximum values (between 20° and 32°) were recorded in zones A and C, while zones B and D exhibited lower shear angles (under 5°). The same trend was observed for the double dome across all three reinforcements (Figure 4). When the shear angles were greater in the hemispherical part of the double dome, this reduced shear can be attributed to the double dome having a radius of 40 mm, compared to the hemisphere’s radius of 50 mm. Almost no shearing was detected in the horizontal half-cylinder section of the double dome.

The triple-point geometries are based on inclined planes meeting at the triple points. Most deformations of the prism occurred on the vertical plane faces, with symmetrical measures between them. There was no noticeable shearing on the lateral inclined planes. On each vertical plane face, the shearing occurs in two opposite directions (shear angles with opposite signs) on the two sides (A with B and C with D), separated by a symmetry axis passing through the triple point. This causes in-plane curvature due to the shift in yarn directions. The maximum shear angles shown in Figure 5a are in absolute values. Shearing ranged from 32° to 43° for the plain weave and from 34° to 38° for the Interlock. The results for the Interlock are consistent with those obtained by Allaoui et al. with this geometry [21,26], the NCF shearing was not symmetrical; it reached as high as 37° on one side (Areas A and C) and as low as 15° (Areas B and D) on the other (Figure 5b). For all tested configurations, the measured shear angles were more significant than those obtained in the hemisphere and double dome preforms, implying that the prism is more severe.

The shearing observed on the tetrahedron appeared on three plane faces. Two depth levels were measured and shown in the tables (Figure 6). For every face, the shearing occurred in two opposite directions on the two triangular subareas delimited by the lines on the top view of the interlock preform. A symmetry was observed in the measured behaviour of plain weave and Interlock along the axis, reaching values close to 30° for woven fabrics. On faces B and C, shear was higher in the subareas c”, b” than c’ b’. The highest shearing for the plain weave and Interlock was found on planes B and C in the subareas b” and c”. Once again, the measured shear on the Interlock is consistent with the literature [26]. The NCF was sheared only on two subareas, a” and c”, with maximum angles measured of 47° and 28°. There was almost no shear for the rest of the preform. Studies have shown that, in some cases, NCF may exhibit different behaviours for positive or negative shear [38,39]. In one direction, shear is dictated by the tensile behaviour of the stitch, resulting in higher shear stiffness. When subjected to compression in the opposite direction, friction determines the resistance, leading to lower shear stiffness. While woven fabric relies on the cohesion between yarns, the behaviour of NCF depends on stitching structure.

Given the maximum shear values measured, which remain globally lower than those obtained on the prismatic preform, it can be concluded that the tetrahedron experiences less severe shearing than the prism.

The highest shearing angles were recorded at the four corners of the cube preform for each depth level (Figure 7). These angles reached 65° for the plain weave and 68° for the Interlock. Shearing was concentrated at the corners along the edges, increasing as it moved towards the bottom. Shear was absent from the other surfaces of the shape. The NCF for this preform, which had two high shearing angles, reached 71° at two opposite corners where the stitching was perpendicular to the edge (portions A and C) and two lower angles of 50° at the corners where the stitching was parallel to the edge (portions B and D). The additional length created by adding depth generates compression strain, which the shear accommodates to adapt to the shape at the corners, reaching its maximum. This area is most likely to produce wrinkles and surpass the locking angles of the fabrics [8,22,40], e.g., for the Interlock with an angle of 55–60°. However, the preforms do not exhibit any wrinkling areas (where there is contact with the punch) as they have been cancelled by choosing to use BHs [16,26].

The plain weave exhibited higher shear angles across the hemisphere, the double dome, and regions A and B of the tetrahedron and the prism, while NCF was highest for area C of the tetrahedron, the cube corner, and the interlock fabric. It has been observed that the plain weave loses its dominance in the highest shearing when sliding occurs near the sheared area. The other fabrics continue to shear at higher angles, while the plain weave is interrupted by sliding of the yarns.

The superior properties of the other two fabrics are aided by the interlacing of the warp yarns with three weft yarn layers in the case of the Interlock, and by the stitching that helps to limit movement between the two layers in the NCF. These results are consistent with the classification of reinforcement behaviour according to the architecture [8], based on shear [17] and bending stiffnesses [41]. The high shears for NCF occur when both yarn networks have rotations toward an axis that is perpendicular to the seams (areas a’ and c” of the tetrahedron and areas A and C of the other geometries), which can be attributed to the effect of the stitching.

**Figure 8:**
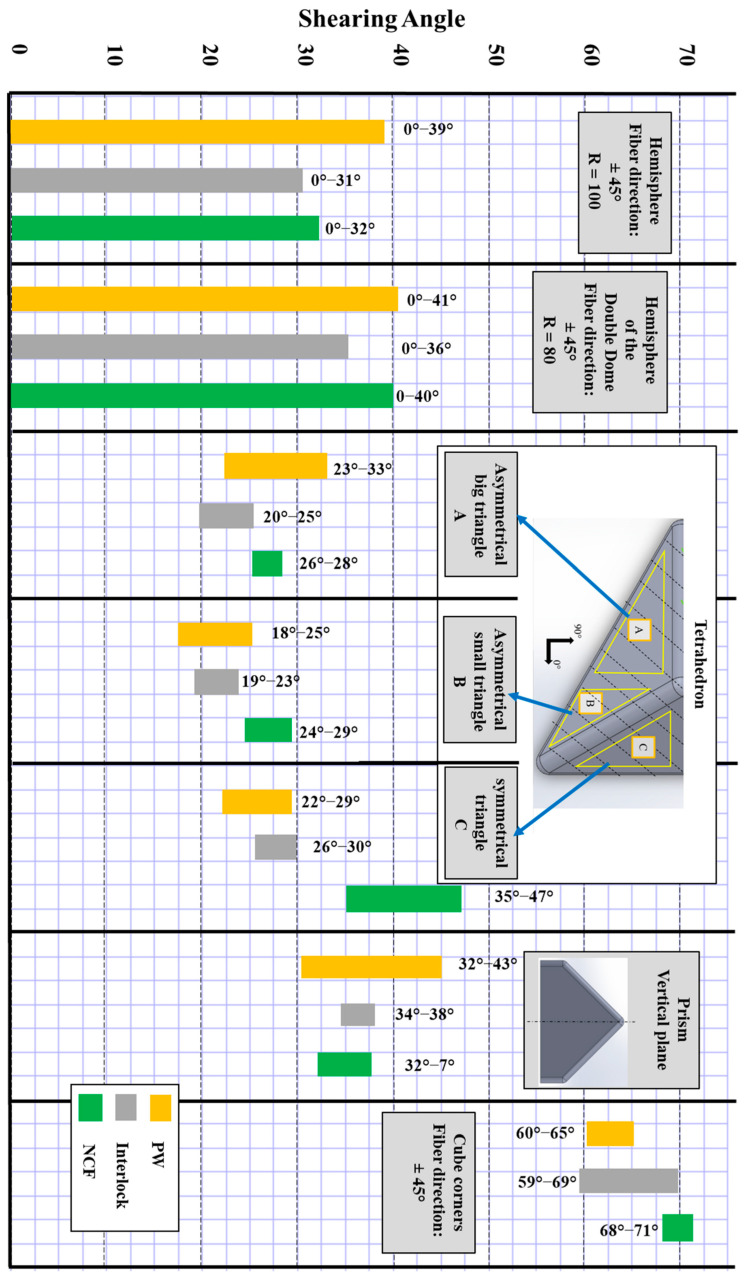
Shearing angle ranges by location measured on the five geometries preforms. Adapted from Ref. [35].

To analyse the effect of punch geometry on shear behaviour, we summarised the measured shear angles and created a classification based on the angle ranges of these facets or parts, as shown in Figure 8. The initial facets are the hemispheric parts in the hemisphere geometry and the double dome geometry, which have different diameters for the latter. The shearing in these facets was generated following the direction of the fabric at ±45°, increasing its amplitude from the top centre to the base of the hemispherical part. Three distinctive zones were identified for the tetrahedron geometry, each with a different range of shearing. The perpendicular plane of the prism is the only area where shearing occurs, as the two inclined planes did not show any significant shearing. Lastly, an interesting detail is the corner of the cube. This cube part triggered the highest shearing found among the five geometries. Therefore, two different features were considered when analysing the various facets or parts: the amplitude and the range of shearing angle values for each part. The hemisphere offers a wider range of angles, from 0° to 40°, depending on the fabric and diameter. This is made possible by the evolution of the radius of curvature from the centre of the hemisphere to the base, as seen in the double dome. This clarifies why many studies focus on the geometries of the hemisphere and double dome. The continuous spectrum of shear angles resulting from this geometry allows for comparison of the shearing behaviour of reinforcements over a broad range. However, the maximum angle of 40° is not sufficient to reach the locking angles.

The tetrahedron, the prism, and the cube have four different shearing ranges, which are more limited than those produced by the hemisphere. The tetrahedron’s shearing amplitude mainly falls between 20° and 33° in the three areas with the three fabrics (except for the NCF in one area), falling within the shear range generated by the hemisphere. The shearing variation observed in the perpendicular plane of the prism is also limited but slightly higher than that of the hemisphere, ranging from 32° to 43°. The highest angles were recorded at the corners of the cube, with shear angles ranging from 59° to 71°, where the amplitude is highly dependent on depth. However, the tetrahedral preform was the most effective in distinguishing the reinforcements, as the differences in shear-induced deformation between various reinforcements at the same location were most significant (a maximum difference of 10° for the hemisphere and 18° for the tetrahedron).

Therefore, these triple-point geometries do not permit wide and continuous shear angle ranges. However, they offer the advantage of producing high induced shear angles, which can stress the reinforcements beyond their locking angles. The most extreme in this regard is the cube, followed by the prism. Therefore, their application in studying formability based on shearing behaviour must be complemented with other geometries (such as the hemisphere) to encompass the entire possible range of angles for reinforcements. Similarly, the geometric singularities of these geometries, like the edges with small radii and the triple point, highlight other phenomena, including mesoscopic defects.

### 3.2. Defects

During the forming process, defects emerge. Different fabric behaviours and deformation mechanisms significantly influence the appearance of defects. A phenomenon is referred to as a defect when deformation alters the fabric’s structure, which may lead to a decline in its mechanical properties. Defects can occur at the yarn level or the fabric level (from multilayers to monolayers) and may involve out-of-plane or in-plane deformation. In this study, defects were identified, located, and quantified by proposing measurement factors related to the physical aspects affected by the defects.

Two types of defects were identified. The first is slippage, a flaw specific to plain weave, attributable to the fabric’s weak cohesion. The second defect is buckling, observed in all three fabrics. Other common defects, such as wrinkling, did not occur in any of the shapes or fabrics, which was expected since the setting was designed to prevent wrinkles from forming.

#### 3.2.1. Slippage

**Figure 9:**
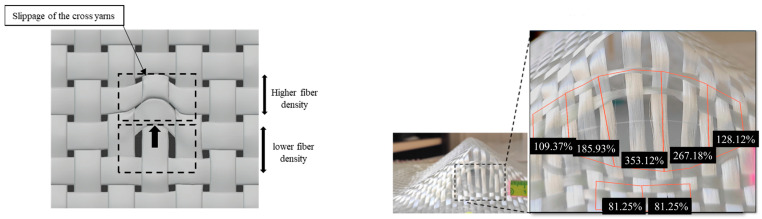
The slippage defect on the plain weave fabric. Adapted from Ref. [35].

Slippage is when the yarn shifts its position in a transversal direction from its axis, creating an in-plane curve. This curvature disrupts the fabric’s meso-structure and creates two distinct areas. The first is an area with high fibre density located where it has slipped, and the second has a low density (Figure 9).

It is crucial to consider this defect because it creates a local weakness in the composite structure, impacting its mechanical properties. Several factors, such as the geometry’s features, process parameters, and fabric behaviours, contribute to the development of slippage [42].

Conventionally, defects are assessed by their surface extent and amplitude. Since slippage causes regions with low fibre density, it is appropriate to include measurements that capture this aspect. Therefore, slippage was characterised using parameters that reflect its amplitude and severity. First, the unit cells (UC) subjected to the maximum change were identified, and their dimensional increase was measured. The variation in each unit cell’s area was calculated and expressed relative to the initial area of the elementary cell. The maximum value (UC max%) attained by this parameter for each geometry is shown in Table 2. This is the parameter that can be associated with the loss of mechanical performance at the local scale, using analytical and numerical models, for example, and thereby indicates the local severity of defects.

The number of locations where the slippage appeared was counted for each preform. This parameter can be linked to the effect of the defect on the composite’s mechanical performance at the macroscopic level. Two other parameters were measured to quantify the expansion and change in the unit cell dimensions. The first parameter, “The average number of UCs defected per location,” represents the average number of affected unit cells counted for each defective zone. The cell was considered defective when there was more than a 10% change. The second parameter, defined as the “average density per location,” is the average decrease in unit cells compared to the initial number occupying the same surface area. These two parameters can explain the severity of the defect in the composite’s behaviour at the macroscopic level.

**Figure 10:**
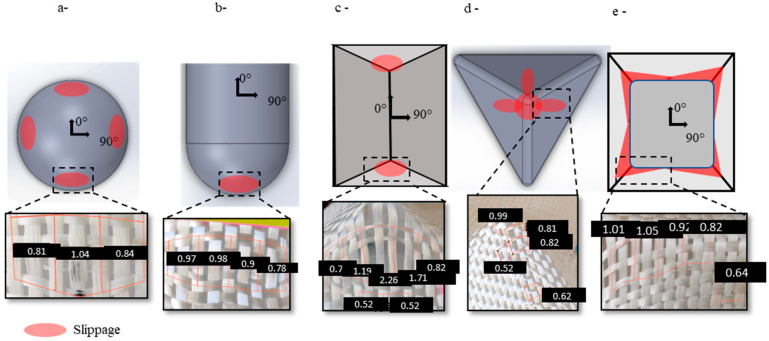
The slippage defects location with the area of the unit cell in cm^2^ on: (**a**) hemisphere, (**b**) double dome, (**c**) prism, (**d**) tetrahedron, (**e**) cube. Adapted from Ref. [35].

In the hemisphere, slippage occurred at the bottom of the preform in four locations following the yarn network directions (0° and 90°) (Figure 10a). It resulted in a maximum 162% increase in the surface area of the most deformed cell, indicating a 62% increase in area (Table 2). This defect affected an average of 6.5 unit cells per location and reduced fibre density by 23.07%. In the double dome at 0° (Figure 10b), slippage occurred at two locations and was slightly less severe, with lower surface damage compared to the hemisphere. The curvature of the hemisphere, combined with the two half-hemispheres in the double dome design, caused variation in the fabric length that needed to be accommodated. This generated in-plane tensile stress, and when this stress exceeded the capacity of inter-yarn cohesion, it led to loss of contact and yarn slippage. In the prism geometry, slippage occurred at the top middle of the vertical plane, near the triple point (Figure 10c). It reached a maximum increase of 353% in the unit cell surface, damaging an average of 19 unit cells in two locations of the preforms. It reduced the fibre density by 47%. The presence of the triple point caused a variation in tension. The yarns passing through the triple point experienced the most significant tension, which decreased further from the centre of the vertical face. This caused the lateral warp yarns to shift until the forces at the interlacing of the two yarn networks locally exceeded the cohesion of the reinforcement, leading to slippage. The in-plane tensile stress was initially resisted by yarn-to-yarn friction, allowing the yarns to stay in contact. However, when preforming reached a certain depth, the tensile stress surpassed the friction due to fabric cohesion; subsequently, slippage defects appeared.

Similar phenomena were observed with the tetrahedron planes, where the slippage occurred at the top of the triple point and along the upper centre of the three inclined planes (Figure 10d). The maximum surface change in the unit cell reached 154%, affecting an average of 4.6 unit cells per location. The slippage created a gap at the triple point and lowered the local density by 35%.

The slippage on the cube occurred at the four triple points on each of the two vertical planes (Figure 10e), and it decreased as the distance from the edge increased. The UC surface change reached 164%, leading to a 37% reduction in density. No sliding was observed on the upper plane of the cube, where the fabric initially contacts the reinforcement and becomes almost integral to it. A similar phenomenon was also seen with the prism and the tetrahedron. Therefore, slippage only took place on inclined or vertical faces.

**Figure 11:**
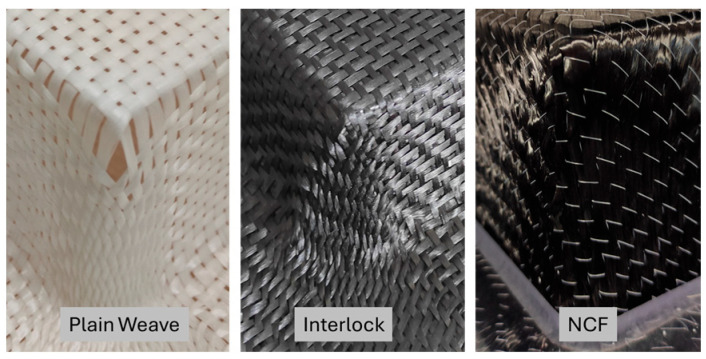
The reinforcement at the corner of the cube, the slippage observation.

Thus, slippage occurred in all the preforms made with the plain weave fabric, unlike the other reinforcements, where no slippage was observed (Figure 11). The geometry’s severity and the test conditions also influence the appearance of this defect. The triple-point geometries produced more severe defects. The prismatic geometry results in the greatest extent and severity of slippage. However, the highest number of defect locations was observed in the cubic preform, which is due to the effect of the cube’s four triple points.

#### 3.2.2. Buckling

**Figure 12:**
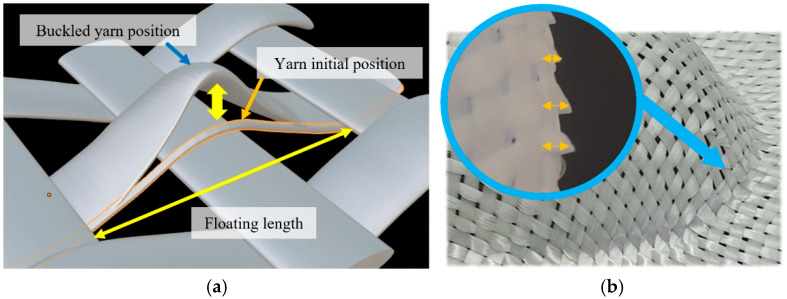
(**a**) tow buckling illustration. (**b**) The buckling defect on the hemisphere preforms with plain weave fabric. Adapted from Ref. [35].

A buckle defect occurs when the yarn is subjected to a compressive load in the same direction as the fibre axis. This defect involves an out-of-plane deformation on the yarn scale, bending it into a curved shape (Figure 12), which impacts its thickness and changes its mechanical properties locally. The profile of the buckling, including its shape and magnitude, depends on the compressive stress and is greatly influenced by the yarn’s structure, such as its width and yarn-to-yarn distance [43], nature of the fibre, and density.

**Figure 13:**
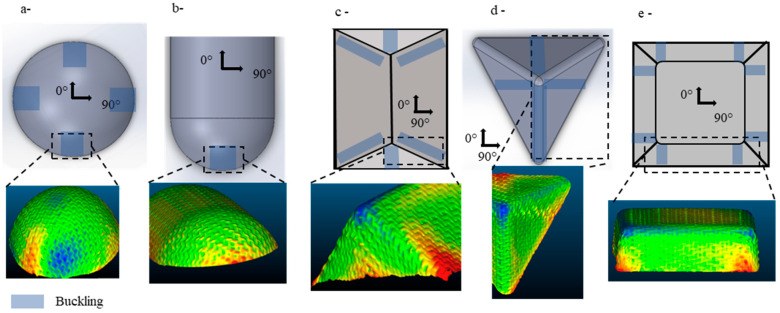
The distance between the outer fabric’s surface and the punch pointing to the buckling locations (**a**) hemisphere. (**b**) double dome. (**c**) prism. (**d**) tetrahedron. (**e**) cube. Adapted from Ref. [35].

This defect was observed across the three fabrics with five preforms at various locations (Figure 13). To characterise this defect and determine its impact, we introduced two categories of parameters. The first relates to the effect of the defect and its severity on a macroscopic level. The first parameter measures the total surface affected in mm^2^. However, when comparing geometries, the defective surface is divided by the total surface area of the preform. The second category concerns the defect’s local dimensions: the buckling amplitude, which is the over-thickness caused by yarn buckling. This reflects the local disturbance of the fabric’s meso-structure and its severity in that specific area.

In the hemisphere preforms, buckles appeared at the directions of 0° and 90° at four locations (Figure 13a). Regarding the double dome, buckles were observed at the base of the two hemispherical parts in the yarn direction (Figure 13b). In these areas, the yarns change curvature as they approach the bottom. Along the width of the yarn, part of it is subjected to compression, while the other part experiences tensile stress. The normal pressure of the cross yarns on the two contact points from both sides creates conditions for the yarns to buckle. Buckling appeared on the two vertical faces of the prism preform, forming a band from the triple point to the bottom (Figure 13c). The yarn passing through the triple point is under high tensile stress, while an in-plane curvature develops because different shearing directions lead to the bending of the transverse yarns, which buckle.

Similarly, buckling occurred on the three inclined planes of the tetrahedron: the yarns passing through the triple point pull the cross yarns, causing the curvature (Figure 13d). Defective areas were observed on all three faces of the preform. The fourth area is located on the edge of the tetrahedron, where the yarns’ directions are parallel to the buckling. The corners of the cube formed buckles at eight locations in total (Figure 13e). These defects arise during the transition from a non-sheared region on the vertical faces to a high-shear region on the vertical planes.

Since the preforming process was conducted under identical settings, it is assumed that the different defect profiles result from the effect of the geometry. Particularly for the plain weave preforms, buckling co-existed with slippage on top of the buckling band. These two defects coincide due to the kinematics and mechanisms involved. As explained above, buckles form when a yarn network is subjected to compression or bending. This stress can be caused by changes in geometrical curvatures, as seen in the case of the hemisphere and the double dome, or by geometric features such as the triple point in other preforms. For example, the latter geometries induce significant tension on the yarns passing through the triple point. This tension causes displacement and acts as a pull-out test by dragging the yarns of the transverse network. The effect depends on the forces and displacements experienced by the pulled yarns, as well as the cohesion of the reinforcement. Therefore, if the reinforcement cohesion is high, the transverse yarns are pulled and bent, creating buckles where the deflection is most significant. If cohesion is low, the pulled yarns slide freely without dragging the transverse yarns.

There is an intermediate situation where these two defects can coexist. This occurs when fabric cohesion is low or diminishes during the shaping process. When the yarns passing through the triple point are pulled in this configuration, they draw the transverse yarns along with them. This leads to the initial formation of loops, which are characterised by out-of-plane buckling and rotation (Figure 12). The rotation of the roving reduces the contact surfaces locally at the point of interlocking with transverse yarns, resulting in a decrease in cohesive forces. These forces depend on these contact surfaces, the friction between yarns, and the tension exerted by the BHs in the transverse direction. When the forces applied to the yarns passing through the triple point exceed the local resisting forces of cohesion, slippage occurs in a stick-slip manner. In fact, as the yarns slide, they reach positions, and again, as the cohesive force increases, causing them to slip again.

**Figure 14:**
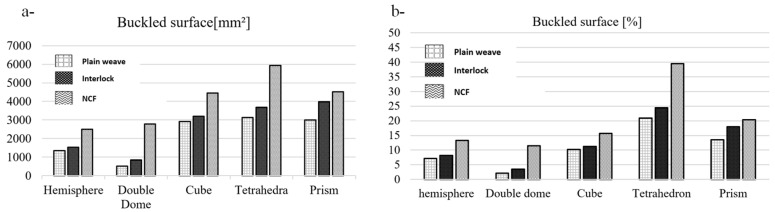
(**a**) The total buckled surfaces of the preforms [mm^2^] (**b**) The total buckled surfaces compared to the total preform surface in %. Adapted from Ref. [35].

Among the five fabrics, buckling affected the NCF the most, reaching over 6000 mm^2^ in the tetrahedron preform and approximately 2500 mm^2^ for the hemisphere (Figure 14a). Unlike the other two woven fabrics, the NCF’s structure consists of two fibre layers, not separate yarns. Each layer contains fibres aligned in the same direction, with two different orientations. It is stitched to maintain the structure. Hence, cohesion between the fibres within the same layer is low, and the effect of the cross-layer stitching makes it more sensitive to bending. For the Interlock and the plain weave, the buckling surface affected was similar in value for each configuration but consistently slightly higher with the Interlock (Figure 14a). The low buckling on the plain weave can be attributed to the slippage’s coexistence.

When comparing different geometries, the tetrahedron produces the largest surface with buckles, as shown by the NCF. Geometries with a triple point cause the most extensive defects, as they consist of several faces connected by transition zones (radii of curvature), unlike the hemisphere and the double dome. However, classifying these geometries based on the surface remains challenging.

To compare the shapes, the size of the final preforms must be considered. When we divided the surface with buckle defects by the total surface of each preform, we obtained the results shown in Figure 13b. These results reveal that the tetrahedron had the highest buckled surface, with over 20% across all three fabrics, reaching as much as 39% with the NCF. This occurs because the geometry of the tetrahedron consists of three inclined faces, not four like the cube and prism, but shear affects all of them. Additionally, the total surface area of the preform is smaller than that of the others and increases with loop zone length, resulting in a larger defective surface. In terms of severity, it is followed by the prism, then the cube. The cube’s geometry at the triple point produces the fewest buckles relative to the total surface area of the preform. This is due to the defects being confined to the edges of the punch. If we alter the relative position of the reinforcement with the cubic punch, the buckles will appear on the faces rather than the edges, leading to an increase in the defective surface area.

**Figure 15:**
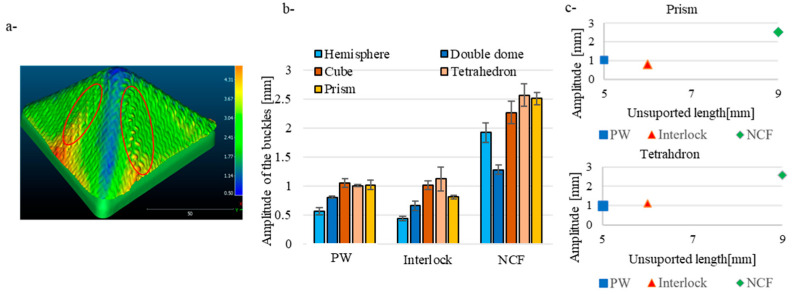
(**a**) 3D scan and the distance between the outer surface of the fabric and the punch (mm). (**b**) The amplitude variation in the buckling defect on the five geometries. (**c**) The amplitude variation in the buckling defect function of the unsupported length of the yarns for the prism and the tetrahedron. Adapted from Ref. [35].

The second category of parameters, which measures the amplitude variation in the yarn’s curvature, indicates the nature of this defect. A scan was performed using a laser 3D scanner to reconstruct the outer surface of the preform (Figure 15a). Subsequently, on the same reference, the preform was removed to scan the outer surface of the punch, and the distance was calculated by projecting the fabric surface onto the punch surface. A map illustrating the variation in the preform’s thickness is obtained (Figure 15a). The results highlight the buckling zone, where the highest distances are observed.

The results shown in Figure 15b illustrate the buckling amplitude for three types of fabrics classified into five geometries. The NCF fabric exhibited a higher buckling amplitude than the woven fabrics, ranging from 2 to 2.5 mm, while the plain weave and the Interlock had a range of 0.5 to 1.2 mm. Buckling behaviour largely depends on the material and structure of the yarns, and in this case, in-plane bending generated the compressive load that caused the buckling. The high buckling amplitude of the NCF can be attributed to the fabric meso-architecture. This is reflected in Figure 15c, where the buckle amplitude is plotted as a function of the unsupported length of the yarn, which is the length of the free-floating section of the yarns where buckling is expected. [44]. The results show a similar pattern to those of the other preforms for the prism and the tetrahedron. The figure clearly indicates that the buckle defect amplitude heavily depends on the yarn’s unsupported lengths. Concerning the effect of geometry, the only trend observed is that triple-point geometries produce higher amplitude buckles than the hemisphere or double dome.

## 4. Conclusions

This study confirms the influence of punch geometry on fabric formability behaviours, such as the shear and defect development during preforming. The five selected geometries generated distinct ranges and distributions of shear angles, highlighting the crucial role of curvature and facet orientation. Double-curved geometries, such as the hemisphere and double dome, enabled broad and continuous shear ranges with comparatively fewer and minor defects. In contrast, the triple-point geometries, the tetrahedron and prism, restricted the shear window but allowed shear angles to exceed the reinforcement’s locking angle due to the orientation of sharp geometric features. These geometries also produced the most significant mesoscopic defects, particularly buckling, with the tetrahedron showing the most pronounced extent. The cube exhibited the highest shear angles among all geometries. Overall, the comparison demonstrates that each geometry triggers specific deformation mechanisms and defect patterns, underlining the importance of selecting a suitable punch shape when evaluating reinforcement formability.

The three reinforcements exhibited distinct behaviours in response to each geometric detail, demonstrating that material architecture dictates both the shear and defect formation. Differences in yarn structure and unsupported yarn length significantly influenced the magnitude and location of buckling, with compressive loads and in-plane bending playing a dominant role in amplitude variations. Slippage defects were observed exclusively in the plain-weave reinforcement. They occurred in the same regions as buckling, producing the highest local density variations in the prism geometry due to the fabric’s low cohesion.

When comparing all geometries, each fabric showed specific advantages and limitations, confirming that no single reinforcement can display a wide range of behaviours. However, the results obtained here can serve as a reference to help narrow down the choice of geometries. By identifying which shapes trigger particular mechanisms, the need for extensive experimental campaigns can be reduced. This also emphasises the importance of focusing on a small number of representative geometries or even developing an optimised geometry that includes the most relevant details. Such an approach would offer an efficient tool for differentiating between reinforcements based on their formability limits while minimising testing efforts.

## Figures and Tables

**Table 1:** Fabrics’ type and properties. Adapted from Ref. [35].

Plain Weave	Interlock (G1151)	NCF (Non-Grimp Fabric)
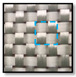	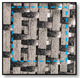	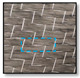
Material: glass fibre Thickness: 0.55 mm Density: 600 Gr/mm^2^ Unit-cell surface: 8 mm × 8 mm Fibres orientation: 0°/90°	Material: carbon fibre Thickness: 0.62 mm Density: 630 Gr/mm^2^ Unit-cell surface: 20 mm× 15 mm Fibres orientation: 0°/90	Material: carbon fibre Thickness:0.8 mm (2 layers) Density: 600 Gr/mm^2^ Unit-cell surface: 9 mm× 5 mm Fibres orientation: +45°/−45°

**Table 2:** The slippage defect of the woven glass fabric (plain weave). Adapted from Ref. [35].

	UC Max (%)	Average UCs Defected per Location	Average Density per Location in %	Number of Defect Locations
Hemisphere	162	6.5	−23.07	4
Double Dome	153	6.45	−22.48	2
Tetrahedra	154.6	4.64	−35.34	4
Cube	164.04	4.17	−37	8
Prism	353.12	18.89	−47.06	2

## Data Availability

The original contributions presented in this study are included in the article. Further inquiries can be directed to the corresponding author.

## Abbreviations

The following abbreviations are used in this manuscript:

LCM	liquid composite moulding processes
BH	Blank holders
NCF	Non-Crimp Fabric
UC	Unit cell
PW	Plain weave
